# Effect of Green Tea on the Level of Salivary Interleukin-1 Beta in Patients with Chronic Periodontitis: A Randomized Clinical Trial

**DOI:** 10.1155/2022/8992313

**Published:** 2022-06-06

**Authors:** Gita Rezvani, Ferial Taleghani, Maryam Valizadeh

**Affiliations:** ^1^Department of Oral & Maxillofacial Pathology, Faculty of Dentistry, Shahed University, Tehran, Iran; ^2^Department of Periodontology, Faculty of Dentistry, Shahed University, Tehran, Iran; ^3^Faculty of Dentistry, Mashhad University of Medical Sciences, Mashhad, Iran

## Abstract

**Aim:**

Interleukin-1 beta (IL-1*β*) is one of the major biomarkers involved in the pathogenesis of chronic periodontitis. The aim of this study was to evaluate the changes in salivary IL-1*β* concentration in patients with chronic periodontitis following daily consumption of green tea.

**Methods and Materials:**

Thirty patients with an average age of 45.8 years suffering from chronic periodontitis were randomly assigned into 2 groups (i.e., experimental and control groups). Besides receiving phase 1 periodontal treatment (scaling and root planning (SRP)), the experimental group drank green tea for a period of 6 weeks. To measure the concentration of salivary IL-1*β*, saliva samples were taken from both groups at 2 time points, i.e., prior to SRP (time point 1 (T0)) and after 6 weeks (time point 2 (T1)). The nonparametric Wilcoxon test was used to examine and compare the changes in the concentration of salivary IL-1*β* in each group relevant to the 2 time points (T0 and T1). Data were submitted to statistical analysis.

**Results:**

At the end of the study period, a significant reduction (*P*=0.0001) in the concentration of salivary IL-1*β* was observed in the experimental group (*A*). As for the control group (*B*), however, there was no significant change (*P*=0.307) in the concentration of salivary IL-1*β* after 6 weeks following phase 1 periodontal treatment.

**Conclusion:**

Green tea supplementation, in addition to SRP, may reduce salivary IL-1*β* levels in patients with chronic periodontitis for a period of 6 weeks.

## 1. Introduction

Periodontitis is inflammation of the tissues that support the teeth with progressive attachment loss and bone destruction. In susceptible individuals, the imbalance between the inflammatory and immune pathways leads to chronic inflammation, tissue destruction, and periodontitis. Many molecular/cellular events and cytokine cascades are involved in this pathway [1].

Interleukin-1 beta (IL-1*β*) is one of the most important cytokines in causing chronic periodontitis, which is the pioneer of other cytokines in the process of inflammation. In a review study, Fróes et al. also showed that this cytokine played an important role not only in the onset of pathogenesis but also in the development of chronic periodontitis [2].

Green tea is made from the leaves of the *Camellia sinensis* plant belonging to the Theaceae family. The decoction of this plant is one of the most popular drinks around the world [3].

It seems that the consumption of green tea can influence the biological environment of the periodontium, and due to its anti-inflammatory property, it can reduce the number of immune mediators produced in response to pathogens [4]. Also, the antimicrobial property of green tea can positively impact the microbial flora of the periodontium, which could lead to a further reduction in inflammatory biomarkers [5].

As concluded in a previous study [6], the consumption of green tea has a positive effect on improving the clinical indices of patients suffering from chronic periodontitis. According to the findings of that study, the inclusion of drinking green tea in the daily diet for a period of 6 weeks following the nonsurgical periodontal treatment can considerably reduce the depth of the periodontal pocket and the bleeding index.

Given the fact that inflammatory cytokines (which are produced as a result of host immune responses to existing pathogenic bacteria caused by chronic periodontitis) are the main factors in the pathogenesis of this disease [5] and that the anti-inflammatory effect of green tea has been confirmed by a series of studies [4, 5], a hypothesis is put forward, that is, it is possible that the improvement in the clinical indices following the consumption of green tea, as observed in previous study [6], occurs as a result of the favorable systemic effect of this substance on the formation of inflammatory cytokines in the periodontium of the subjects.

Accordingly, the present study was designed to evaluate how effective the consumption of green tea can be in terms of reducing the IL-1*β* concentration level in the saliva of patients with chronic periodontitis. Based on this, the statistical null hypothesis is that with daily consumption of green tea for 6 weeks, there will be no significant change in salivary interleukin levels.

## 2. Materials and Methods

### 2.1. Trial Design and Ethics

The present study followed the CONSORT (Consolidated Standards of Reporting Trials) guidelines and was performed in accordance with the World Medical Association Declaration of Helsinki. This article reported the second phase of a randomized controlled, double-blind clinical trial study registered on the Iranian Registry of Clinical Trials website (code: IRCT2015113025296N1). The Ethics Committee in Research of Shahed University approved this study in reference to letter no. 41/219227.

### 2.2. Participants

Fifty patients (42–54 years of age) were selected from the Periodontology Department of Shahed University from January 2017 to May 2017. Inclusion criteria for patients with chronic periodontitis should include all 4 of the following criteria [4]:

(i). Willing to participate in the study and signing the informed consent form
(ii). Presence of at least 2 teeth per quadrant with ≥5 mm probing depth
(iii). Presence of bleeding on probing (BOP) in ≥5 mm and
(iv). Clinical attachment loss (CAL) > 3 mm

Exclusion criteria were as follows:

(i). Systemic disease
(ii). Pregnancy and breastfeeding
(iii). Obtaining periodontal treatment in the last 6 months
(iv). Smoking and drinking alcoholic beverages
(v). Taking nonsteroidal anti-inflammatory drugs (NSAIDs) and antibiotics in the past 6 months
(vi). Drinking more than 1 cup of green tea every week and
(vii). Having fixed orthodontic appliance

Based on the new classification of periodontal diseases (2017), the periodontal patients participating in the present study are in accordance with stages 1 and 2 and grade A [7].

### 2.3. Intervention

Patients were divided into experimental (*A*) and control (*B*) groups. Informed consent was obtained from all the participants prior to their participation in this study.

Saliva samples were taken from all 42 patients prior to the scaling and root planning (SRP) procedure. The samples were taken during an interval less than 1 hour after breakfast. The patients were asked to wash their mouths with tap water before taking samples and spit their saliva into sample containers. Then, 0.5 mL of each sample was transferred to a microtube using a sterile syringe and immediately stored in an ice container. Eventually, the samples were stored in ice-containing boxes and transferred into a freezer at −70°C. All patients received SRP treatment by an operator (final year dental student) under the supervision of a periodontist; they were given instructions on complete oral hygiene, including a brushing technique (modified bass) and flossing. The method and duration of using green tea verbally and in writing were explained to the experimental group in the same session.

Commercial green tea leaves (Momtaz Green Tea, Lahijan, Iran) were purchased, and its scientific name was confirmed by a botanist (L48I-Herbarium 8732; *Camellia sinensis* L.).

The experimental group drank green tea for 6 weeks following SRP treatment, but the control group only received SRP treatment. The consumption instruction was to drink 2 cups a day (morning and night) after brushing; each cup contained 25 g of green tea leaves [6].

One of the operators (M. V.) contacted participants by phone to ensure that the experimental group drank green tea according to the instructions and that both groups adhered to personal oral hygiene according to the instructions. Patients in both groups should notify the authors if they receive medication or dental treatment during the 6 weeks in order to be excluded from the study.

After 6 weeks, both groups were recalled to the clinic, and saliva samples were taken the same way as before the intervention.

### 2.4. Outcome

The IL-1*β* concentration level of samples taken from both groups before and after the intervention was measured using a DuoSet ELISA kit (R&D Systems, Thermo Fisher Scientific, Canada) and applying an indirect enzyme-linked immunosorbent assay (ELISA) technique. The results were reported as picogram per milliliter (pg/mL). The practitioner who conducted the ELISA tests was blinded to the intervention.

### 2.5. Sample Size

**Figure 1:**
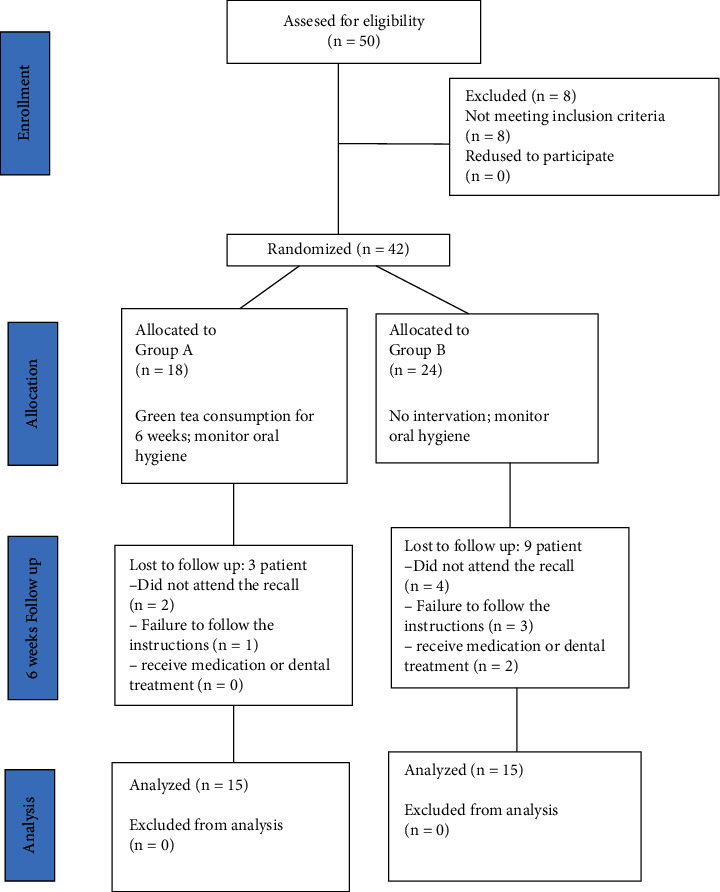
CONSORT flowchart of participants' trial steps.

The sample size was calculated by considering the 95% CI, 80% power, *α* equals 0.05 (standard deviation), 15% success rate in the control group, and 60% success rate in the experimental group. A prototype of 15 items per group was identified, to which 20% was added to compensate for the potential drop, resulting in a final sample of 18 items per group. The calculation was performed using sealed envelope (https://www.sealedenvelope.com/power). Also, according to a similar study [8] and based on our primary outcome (mean difference of IL-1*β* concentration level), 15 items in each group seemed sufficient (Figure 1).

### 2.6. Randomization

One of the staff members of the periodontics department divided the 42 selected patients into 2 groups by producing random numbers 0 (control) and 1 (experimental) using a computer system after SRP treatment. If the patient was in the experimental group, she informed another operator (M. V.) to teach her/him how to drink green tea. Then, the subjects were matched in terms of age and gender. The experimental group had 18 patients (including 6 males and 12 females), and the control group had 24 patients (including 16 males and 8 females).

### 2.7. Blinding

For personal blinding, both persons who performed SRP treatment and measured clinical parameters (final year dental students), as well as the person who collected saliva samples (M. B.), did not know which group each patient belonged to. Only one of the research team members knew which group each participant belonged to (M. V.), who was responsible for delivering specific packages of green tea to the experimental group.

### 2.8. Statistical Analysis

SPSS version 21 (SPSS Inc, Chicago, USA) was used for statistical analysis of the data. The Kolmogorov–Smirnov test revealed that data were not normally distributed. The test was significant for both groups (*P* < 0.00) in the sense that we had to use nonparametric tests to analyze the data. The nonparametric Wilcoxon test was used to examine and compare the changes in the concentration levels of salivary IL-1*β* in each group relevant to the 2 time points (time point 1 (T0), before the treatment and time point 2 (T1), after 6 weeks). The nonparametric Mann–Whitney test was used to study the differences between the 2 groups. *P* values less than 0.05 were considered statistically significant.

## 3. Results

### 3.1. Participant Flow and Baseline Data

At baseline, 50 patients were evaluated for eligibility: 29 females (58%) and 21 males (42%), with a mean ± SD age of 48 ± 1.23 years. Forty-two patients were selected for further investigation. Finally, during the study, 12 patients were excluded from the study (Figure 1). The demographic status of the remaining 30 patients was as follows: the experimental group had 15 patients (9 females and 6 males) with a mean ± SD age of 45.2 ± 2.15 years, and the control group had 15 patients (8 females and 7 males) with a mean ± SD of 46.5 ± 2.32 years (Table 1). All periodontitis patients in our study were in stages 1 and 2 and grade A of periodontal disease classification.

### 3.2. Outcome and Estimation

**Figure 2:**
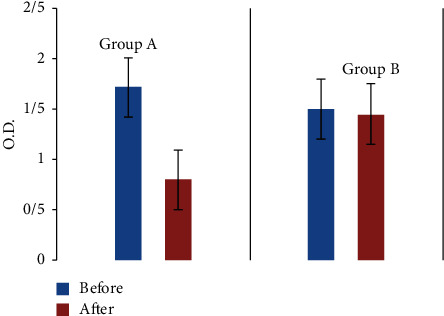
Comparison of optical density (O.D.) values before and after intervention in groups A and B.

Since the optical density (OD) values read on the ELISA reader have a positive correlation with IL-1*β* concentration levels, OD values were used to ease the process of statistical analysis. As given in Table 2, the concentration level of IL-1*β* in the experimental group significantly decreased (*P*=0.001) after intervention. However, the amount of this salivary biomarker in the control group, who merely received the first phase of periodontal treatment, did not change significantly (*P*=0.307). Also, the IL-1*β* concentration level was significantly lower (*P* < 0.001) in the experimental group following the intervention and after 6 weeks (0.92 ± 0.59) compared with the control group (0.05 ± 0.57).  Figure 2 shows the changes in the IL-1*β* concentration in groups A and B.

### 3.3. Harms

We tried to reduce the likelihood of drug interactions by excluding patients who took medicine or received dental treatment during the intervention period (6 weeks). However, during the intervention period (6 weeks), 2 patients in the control group (6.6%) were excluded from the study due to drug use. Also, during the intervention period, 1 patient expressed heartburn after drinking green tea, but he willingly continued to drink green tea.

## 4. Discussion

The present clinical trial study was designed to evaluate the effect of the daily consumption of green tea on periodontal tissues in patients with chronic periodontitis. For this purpose, the changes in the level of the IL-1*β* inflammatory cytokine (which plays a major role in the pathogenesis of chronic periodontitis) were investigated. The results indicate that the daily consumption of 2 cups of green tea for a period of 6 weeks following SRP can significantly reduce the concentration of salivary IL-1*β* in patients with chronic periodontitis compared with the control group.

Periopathogens such as *Fusobacterium nucleatum* and *Porphyromonas gingivalis* have been shown to dysregulate IL-1*β* through a variety of mechanisms [9, 10]. Moreover, it has been established that the concentration level of IL-1*β* in gingival cervical fluid (GCF) from areas affected by periodontal disease is notably high; however, on the condition that the response to treatment is favorable, the IL-1*β* concentration level will decrease in saliva and GCF [11, 12]. A systematic review and meta-analysis study found that although changes in salivary IL-1*β* and matrix metalloproteinase 8 (MMP-8) levels after nonsurgical periodontal treatment were not statistically significant, salivary cytokines could be used to confirm the effect of periodontal treatment or to diagnose periodontal disease [3]. Therefore, at the present time, a particular value is given to this biomarker with regard to assessing the extent of success in the treatment of periodontitis [12, 13].

Nutrients and certain bioactive compounds, such as polyphenols, have been identified as contributing factors to the relief of symptoms in and recovery from periodontitis [14]. Catechin compounds found in green tea, as its primary polyphenolic compounds, are believed to possess antioxidative, anti-inflammatory, and antimicrobial properties, which have been reported to positively impact the clinical parameters in patients with chronic periodontitis [15]. A meta-analysis study (2021) showed no difference in the effectiveness of green tea alone or in combination with SRP to reduce the depth of the pocket. In this meta-analysis study (included 9 studies), 5 clinical parameters were used to evaluate the effect of green tea. Due to short follow-up periods, the accuracy of these included articles is low and very low according to the GRADE tool and clinical parameters and is not reliable to evaluate the response to treatment and start the healing process; accordingly, it seems that using a molecular assay tool is logical [16]. In a similar meta-analysis, Mazur et al. examined the antiperiodontitis and anticaries effects of green tea. The authors concluded that there was insufficient evidence to recommend the use of green tea formula as the first treatment for gingivitis, periodontitis, and caries. In the studies included in this study, only clinical parameters were used [17].

In an animal study, it was reported that the injection or oral administration of catechin in green tea significantly reduced the expression of IL-1*β* (induced by lipopolysaccharides from pathogenic bacteria in the periodontium) and, consequently, the resorption of the alveolar bone [18]. Production of CC chemokine ligand 11 (CCL11) in human gingival fibroblasts (HGFs) is associated with the migration of Th2 lymphocytes in the pathogenesis of the periodontal disease. Treatment with epigallocatechin gallate (EGCG) has been shown to inhibit the production of CCL11 induced by IL-1*β*/IL-4 or IL-4/TNF-*α* [19]. Some studies have shown the local effect of green tea placed in periodontal pockets on reducing periopathogens, such as *P. gingivalis* [20, 21]. In an in vitro study, the effect of green tea catechins on inflammation of periodontal disease was investigated. The results of this study showed that catechin reduced the production of IL-1*β* by lymphocytes and inhibited inflammasomes induced by *P. gingivalis* [22].

It should be noted that due to the fact that “natural” is not always safe, green tea can also be harmful. The most important side effects of green tea are gastrointestinal disorders, especially when consumed on an empty stomach, and hepatotoxicity. However, concentrated, solid, and large doses of green tea (rich in catechins) but not brewed tea or green tea extract products as part of the diet have been shown to cause liver problems. The active ingredients in green tea are not teratogenic, mutagenic, or carcinogenic; however, it is not yet clear whether they can be used during pregnancy and lactation [23, 24].

Recent studies have shown that periodontitis is caused by enhanced interactions between irregular inflammation and a dysbiotic microbiome. Inflammation, in addition to being a consequence of dysbiosis, provides conditions for the selective growth of bacteria. Therefore, host modulation therapies aimed at preventing harmful inflammation and reducing connective tissue and bone loss seem reasonable [25]. These include artificial and biological disease-modifying antirheumatic drugs. Treatment with recombinant human monoclonal antibodies against CD20 (rituximab) and IL-6 receptor (tocilizumab) reduced periodontal inflammation and improved periodontal condition. Studies on the effect of TNF-*α* inhibitors in patients with periodontitis have yielded conflicting results. Recent data suggest that probiotics, including dietary supplements such as *n*-3 fatty acids, have anti-inflammatory benefits when combined with periodontal treatment. Probiotics reduce the production of proinflammatory cytokines/chemokines and increase T cell regulatory cell accumulation [26].

A new treatment introduced by the use of lasers in dentistry is photobiomodulation (PBM) therapy as a supplement to SRP. In a split-mouth design clinical trial comparing the effects of ozone and PBM as a complement to SRP in reducing the clinical parameters of periodontal patients, it was shown that both ozone and PBM are effective adjunctive therapies for SRP, and a slightly better result is expected for the latter in the long-term to reduce the depth of the periodontal pocket [27]. In addition, it has been shown that the use of diode laser with SRP improves clinical parameters by reducing sclerostin (a bone formation inhibitor) levels in the GCF of patients with chronic periodontitis [28].

Nevertheless, there seems to be no study in the literature that evaluates the effect of consuming green tea on the number of pathogenic microbes and the level of salivary inflammatory biomarkers.

The limitations of our study are as follows: the follow-up period was short, and we did not monitor IL-1*β* in GCF and microbial examination of periodontal pocket before and after treatment.

Also, considering that green tea has many active ingredients, it is not clear which substance plays a role in the antiperiodontitis effect; thus, further cell and molecular studies are needed in this regard. In addition, obtaining saliva samples has its limitations, including stimulating saliva secretion, performing the aliquot process to increase the quality of samples that require equipment such as centrifugation, and transferring and storing saliva samples at low temperatures.

Matching conditions, such as oral hygiene status before participating in the study, were done by asking about the frequency of brushing and flossing per day, although this method is not completely valid. Other conditions such as systemic disease, pregnancy or lactation, or medication may affect the outcome; thus, these patients were excluded from the beginning.

Based on the results of the present study and their connection with those achieved in a previous study [6], the systemic consumption of green tea can lead to improved clinical indices in patients with chronic periodontitis by reducing the IL-1*β* level. However, further studies are needed to examine the effect of green tea on other inflammatory cytokines and evaluate the antibacterial property of green tea as a possible contributor to better clinical indices in patients with chronic periodontitis.

## 5. Conclusion

Compared to SRP treatment alone, daily consumption of green tea in combination with SRP treatment can significantly reduce important inflammatory cytokine IL-1*β* in salivary samples. Longer follow-up time and measurement of this marker in GCF may show this difference more and better. It seems that we can help reduce inflammation and improve periodontal conditions by recommending a simple change in daily diet.

Also, more research studies need to be done to determine the role of the changes in other inflammatory mediators in response to the consumption of green tea in periodontitis.

## Figures and Tables

**Table 1:** Demographic information of participants after the final dropout.

	Gender	Age (mean ± SD^*∗*^)
Group A	Female: 9 patients	45.2 (±2/15)
	Male: 6 patients	
Group B	Female: 8 patients	46.5 (±2.32)
	Male: 7 patients	

^*∗*^Standard deviation.

**Table 2:** Comparison of O.D. values before intervention and after 6 weeks in each group as well as comparison of resultant data between the two groups.

Groups	Number	Mean ± SD^*∗*^ (before)	Mean ± SD (after)	*P* value (each group)	^*∗∗*^ *P* value (intergroup)
A	15	1/71 ± 75/0	0.79 ± 0.41	^*∗∗*^0.001	0.0001
B	15	1.49 ± 0.78	1.44 ± 0.63	0.307	

^*∗*^SD, standard deviation. ^*∗∗*^Significant.

## Data Availability

The data used to support the findings of this study are included within the article and are available from the corresponding author upon request.
